# Computed tomography and magnetic resonance imaging of desmoplastic fibroma with simultaneous manifestation in two unusual locations: a case report

**DOI:** 10.1186/1752-1947-5-28

**Published:** 2011-01-24

**Authors:** Konstantinos Stefanidis, Stelios Benakis, Emmanouela Tsatalou, Vasilios Ouranos, Dimitrios Chondros

**Affiliations:** 1 CT and MRI department, Evangelismos Hospital, Ipsilantou 45-47, 10676, Athens, Greece; 2 CT and MRI department, 7thIKA Hospital, Athens, Greece

## Abstract

**Introduction:**

Desmoplastic fibroma is an extremely rare primary benign bone tumor. It occurs most often in the mandible, followed by the femur and pelvis. To the best of our knowledge, fewer than 200 cases have been described in the published literature. Furthermore, this case is the first report of desmoplastic fibroma with simultaneous presentation in two different locations.

**Case presentation:**

We present an unusual case of desmoplastic fibroma in a 56-year-old Caucasian man, who presented to our hospital with lumbar pain. Computed tomography and magnetic resonance imaging were performed, demonstrating two lytic expansile lesions affecting both his left iliac bone and his left sacral wing. Curettage and cortical-cancellous grafting was performed, followed by postoperative computed tomography and magnetic resonance imaging.

**Conclusion:**

Desmoplastic fibroma with unusual and simultaneous manifestations in two different locations has never been reported previously to the best of our knowledge. The purpose of this case report is to present the computed tomography and magnetic resonance imaging features of this rare tumor before and after the surgical treatment. Furthermore, the radiological findings with the description of the characteristics and the clinical presentation of this rare tumor, contribute to the wide spectrum of manifestations of this tumor, in order to recognize it and to have the appropriate management.

## Introduction

Desmoplastic fibroma (DF) of bone is a rare, lytic, locally aggressive but non-metastatic tumor that was first described by Jaffe in 1958 [1]. It is an extremely rare tumor with less than 200 cases in the published literature and with a reported incidence of 0.11% to 0.13% among primary bone tumors [2]. It occurs most often in the first three decades of life and is found equally in men and women [3]. The most common site is the mandible, followed by the femur and pelvis. In this case report we present and analyze the computed tomography (CT) and magnetic resonance imaging (MRI) features of this rare tumor affecting both the left iliac bone and left sacral wing.

## Case presentation

**Figure 1:**
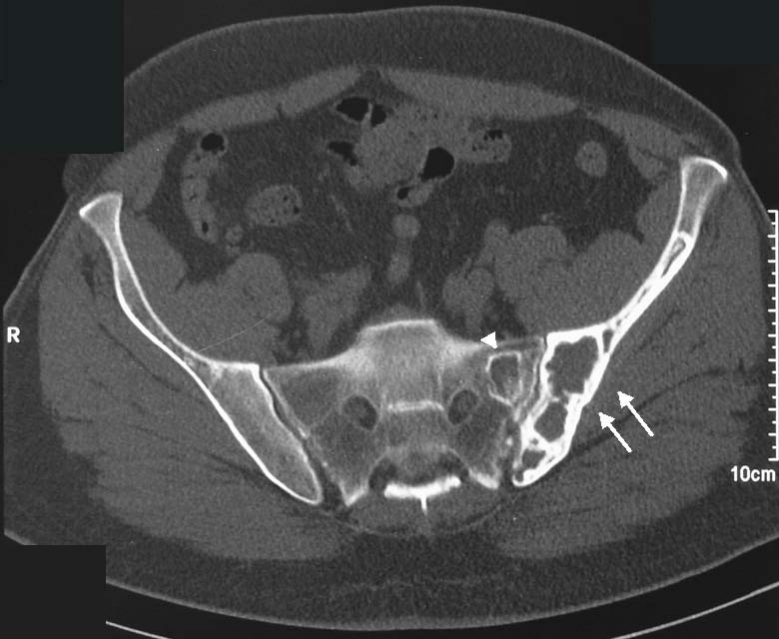
**Axial CT in bone density, demonstrates a lytic expansible lesion with cortical breakthrough at the left iliac bone (arrows) and a smaller one at the left sacral wing at the level of S2 (arrowhead)**.

**Figure 2:**
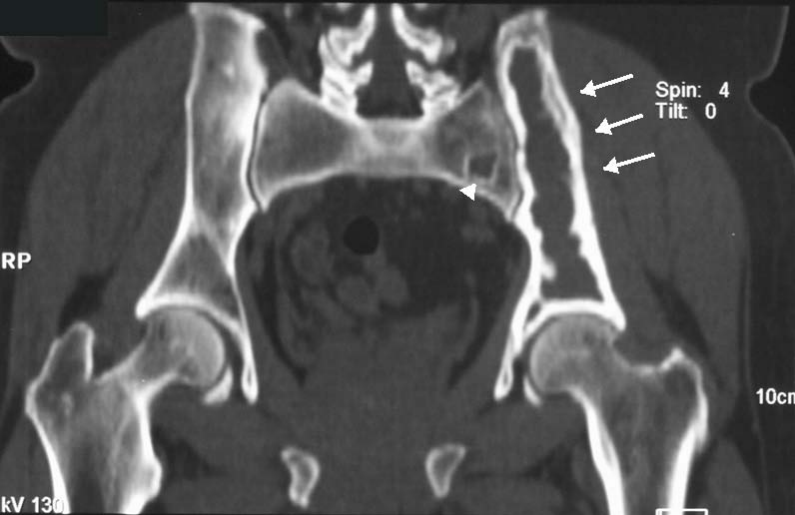
**Coronal CT in bone density, shows the extension of the lytic lesion at the left iliac bone e (arrows) and at the left sacral wing at the level of S2 (arrowhead)**.

**Figure 3:**
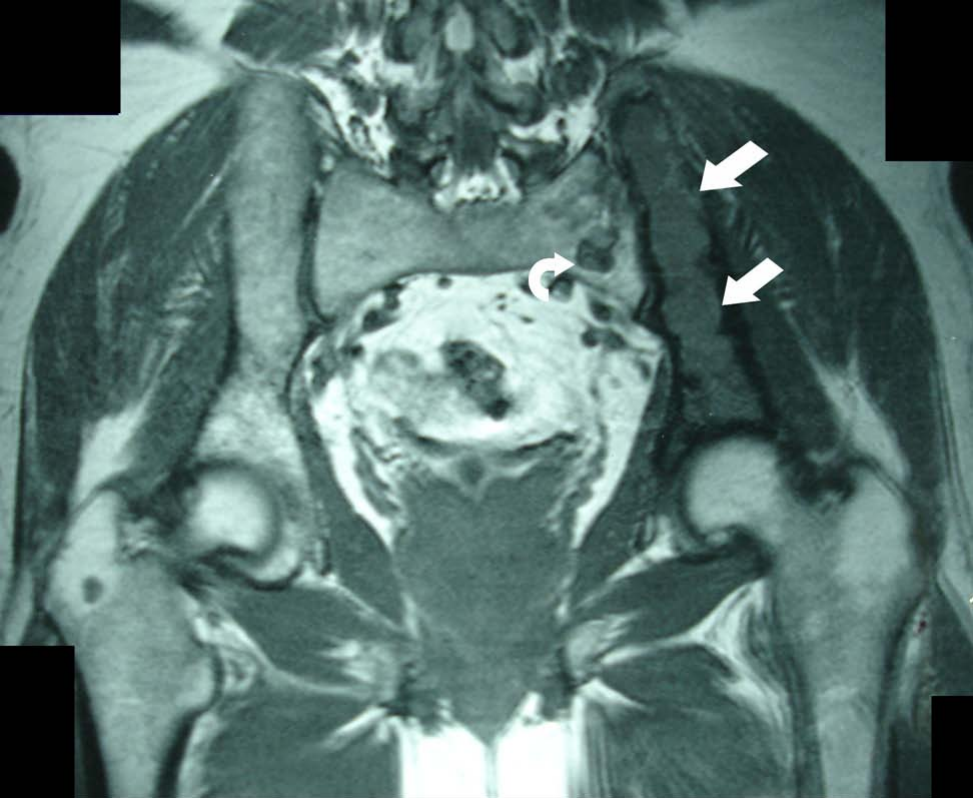
**Coronal T1-weighted MRI demonstrates decreased signal at the left iliac bone (arrows) and at the left sacral wing (curved arrow)**.

**Figure 4:**
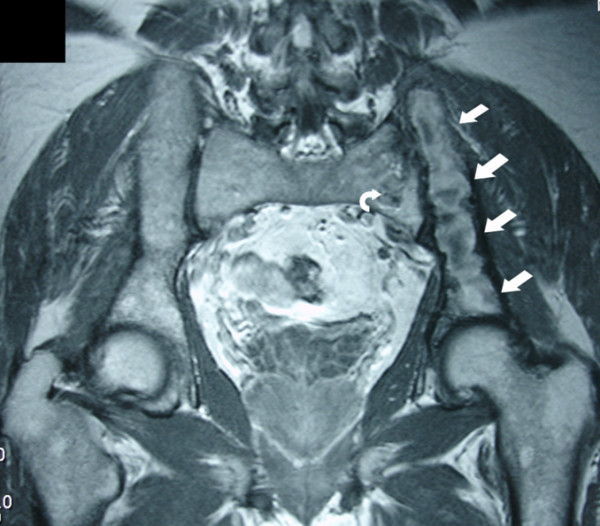
**Post contrast coronal T1-weighted MRI shows a heterogeneous increased signal at the left iliac bone (arrows) and at the left sacral wing**.

**Figure 5:**
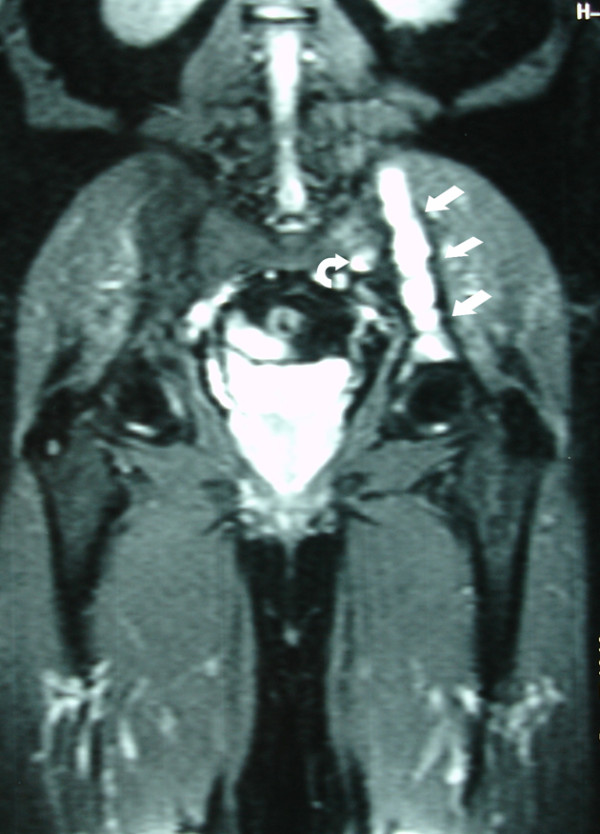
**Coronal STIR.MRI image shows an increased signal at the left iliac bone (arrows) and at the left sacral wing (curved arrow)**.

A 56-year-old Caucasian man, with a history of lumbar pain, presented to our hospital for a CT lumbar spine scan. He had no history of trauma. CT scan demonstrated an osteolytic lesion of the left iliac bone with cortical breakthrough, marginal sclerosis, slight pseudotrabeculation and bone expansion (Figures 1, 2). A smaller lesion with a similar lytic pattern was depicted with smaller extension in the left sacral wing at the level of S2. An MRI scan was performed. On T1-weighted sequences the lesions were of low signal intensity (Figure 3) and on post contrast T1-weighted images the lesions presented with high signal intensity with small areas of intermediate signal intensity (Figure 4). On STIR images the tumor displayed a high signal intensity from the lesions (Figure 5).

**Figure 6:**
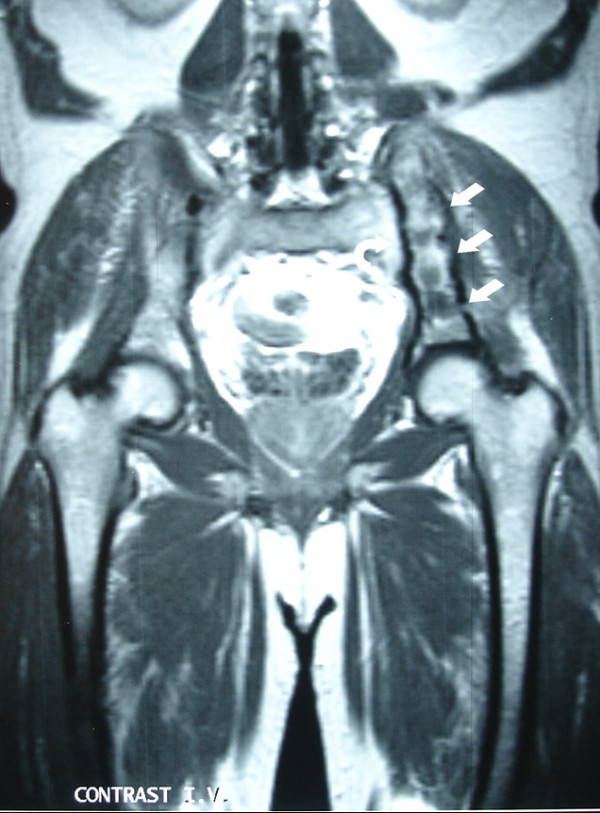
**Postoperative post-contrast coronal T1-weighted MR image demonstrates diminution of the extension and the enhancement of the tumor at the left iliac bone (arrows) and at the left sacral wing (curved arrow)**.

**Figure 7:**
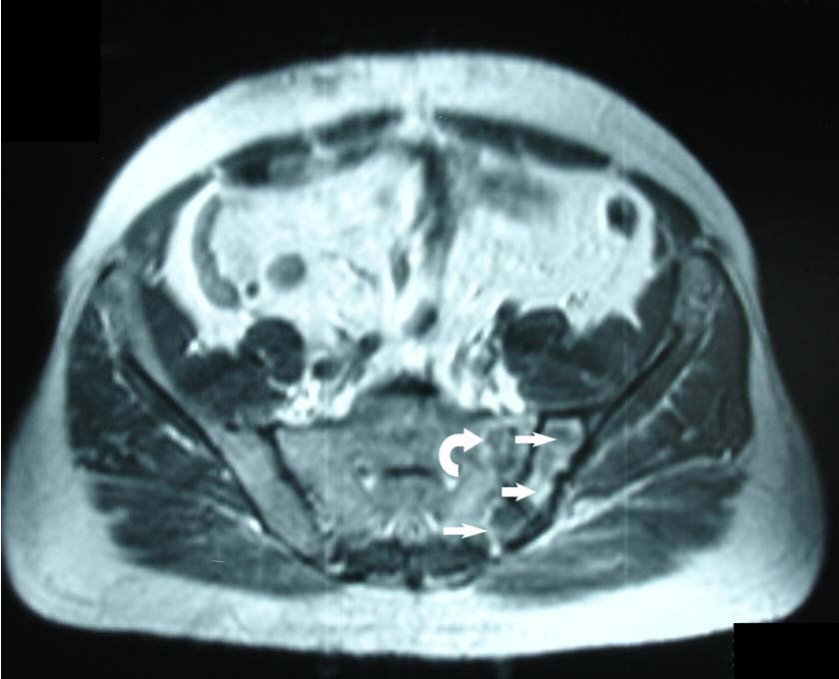
**Post-contrast axial T1-weighted MR image shows the postoperative changes with diminution of the extension and the enhancement of the tumor at the left iliac bone (arrows) and at the left sacral wing (curved arrow)**.

The surgical treatment included curettage and intralesional extension with replacement by cortical-cancellous allograft. The pathological specimen was reported and the histological findings concluded the diagnosis of DF. The surgical management was followed by postoperative CT and MRI, which showed a reduction in size of the lesion (Figures 6, 7). The patient has been monitored for six years without any recurrence.

## Discussion

DF is a rare benign primary bone tumor, histologically similar to the soft tissue desmoid tumor. Microscopic infiltrations of tumor are present beyond the perceived macroscopic margin. In our case, we described a rare bone tumor affecting simultaneously both the left iliac bone and the left sacral wing with interposition of macroscopically normal tissue.

DF is considered an aggressive tumor, with a high incidence of local recurrence after surgical resection (42 to 67%) [4]. Clinical signs of this rare bone tumor are usually nonspecific. Pain and swelling are the predominant symptoms, but some patients may be asymptomatic and the tumor may be an incidental finding. Pathological fractures are reported in 9 to 15% of cases. Treatment of DF of the bone includes curettage, intralesional, marginal or wide resection with or without replacement by allograft, cryosurgery and even amputation in some cases [2,5-7].

Almost any bone can be affected, but DF most often involves the mandible (22%), femur (15%), pelvic bones (13%), radius (12%), and tibia (9%) [2]

DF is a locally aggressive tumor with high recurrence rate and it is important to differentiate it from other bone lytic lesions as there are significant implications regarding surgical treatment. The radiographic appearance of DF is similar to other lytic lesions. Differential diagnosis includes giant cell tumor, aneurysmal and solitary bone cyst, hemangioma, fibrous dysplasia, nonossifying fibroma, and chondromyxoid fibroma.

While CT best illustrates the extent of bone destruction, MRI better visualizes the medullary as well as the soft tissue extension of the tumor. Therefore, CT and MRI are complementary imaging techniques in suspected DF.

## Conclusion

DF of the bone is a rare benign tumor with clinical symptoms that are usually non-specific. It is a benign tumor that should be included in the differential diagnosis list of any lytic bone lesion, especially in young patients. However, it differs from other benign fibrous lesions in that the lesion is very destructive locally and often recurs after incomplete excision. Therefore, despite its rarity, knowledge of the radiological features in correlation with the pathological findings is crucial in the diagnosis and the treatment of this rare tumor.

## Abbreviations

DF: desmoplastic fibroma; CT: computed tomography; MRI: magnetic resonance imaging

## Consent

Written informed consent was obtained from the patient for publication of this

case report and accompanying images. A copy of the written consent is available for review by the Editor-in-Chief of this journal.

## Competing interests

The authors declare that they have no competing interests.

## Authors' contributions

KS prepared the case report and reviewed the literature. SB, ET and DC participated in the conception, design, and data collection and interpretation, analyzed the article and made necessary corrections. All authors read and approved the final manuscript.
